# Sodium-Glucose Cotransporter Inhibition Preserves Apolipoprotein M During Acute Inflammation in Mice and Humans

**DOI:** 10.1016/j.jacadv.2025.101839

**Published:** 2025-06-25

**Authors:** Carla Valenzuela Ripoll, Aynaz Lotfinaghsh, Zhen Guo, Tripti Kumari, Kana N. Miyata, Alireza Sargazi, Mualla Ozcan, Ahmed Diab, Anahita Ataran, Hamidreza Hajirezaei, Omid Rashidi, Wenjing Yu, Yoonje Cho, Attila Kovacs, Carla Weinheimer, Jess Nigro, Olivier Cases, Renata Kozyraki, Jan Oscarsson, Russell Esterline, Moshe Levi, Justin H. Berger, Joel D. Schilling, Rajendra S. Apte, Marco Sardiello, Alexander Peikert, Mikhail Kosiborod, Luigi Adamo, Charlie Lowenstein, Christina Christoffersen, Jaehyung Cho, Ali Javaheri

**Affiliations:** a Washington University School of Medicine, St. Louis, Missouri, USA; b Saint Louis University, St. Louis, Missouri, USA; c INSERM UMRS 1138, Centre de Recherche des Cordeliers, Paris-Diderot University, Paris, France; d BioPharmaceuticals R&D, AstraZeneca, Gothenburg, Sweden; e BioPharmaceuticals R&D, AstraZeneca, Gaithersburg, Maryland, USA; f Georgetown University, Washington, DC, USA; g Department of Cardiology, University Heart Center Graz, Medical University of Graz, Graz, Austria; h St. Luke's Hospital Mid America Heart Institute, Kansas City, Missouri, USA; i University of Missouri-Kansas City, Kansas City, Missouri, USA; j Division of Cardiology, Johns Hopkins University, Baltimore, Maryland, USA; k Department of Clinical Biochemistry, Rigshospitalet and Department of Biomedical Sciences, Copenhagen, Denmark; l John Cochran Veteran Affairs Hospital, St. Louis, Missouri, USA

**Keywords:** apolipoprotein M, endothelial vascular integrity, LRP2, SGLT2

## Abstract

**Background:**

Sodium-glucose cotransporter inhibitors (SGLT2is) reduce inflammation and maintain vascular integrity. Apolipoprotein M (ApoM) is crucial for vascular integrity via sphingosine-1-phosphate (S1P) signaling and is inversely linked with mortality in sepsis and COVID-19.

**Objectives:**

The authors tested if SGLT2i (dapagliflozin [Dapa]) increases ApoM in mice using lipopolysaccharide (LPS) and in humans with COVID-19.

**Methods:**

Diet-induced obese mice (n = 14-15/group), proximal tubule-specific knockout of the multiligand protein receptor Lrp2 (*Lrp2*^*KO*^) mice (n = 5-8/group), *Ly6G-Cre LoxP-STOP-TdTomato* mice (n = 3-5/group), *Apom*^*KO*^ mice (n = 3-5/group), and *Apom*^*TG*^ mice (n = 3-5/group) were randomized to receive either vehicle or Dapa (1.25 mg/kg daily) for 4 days before LPS (10 mg/kg IP). Outcomes included ApoM protein levels (Western and enzyme-linked immunosorbent assay) and intravital microscopy to assess endothelial leak and neutrophil behavior. Plasma samples from ACTIV-4a participants (standard of care, n = 37; standard of care + SGLT2i, n = 15) were analyzed for circulating ApoM by enzyme-linked immunosorbent assay. Statistical analyses included two-way analysis of variance for mice and *t*-test or Mann-Whitney test for humans.

**Results:**

Dapa restored circulating ApoM levels in LPS-treated mice (0.017 vs 0.035 [a.u./μL], *P* = 0.0489) and increased ApoM levels in patients randomized to SGLT2i (0.5240 vs 0.6537 [μM], *P* = 0.0101). LRP2 knockout blocked Dapa's effect on ApoM. In vitro, Dapa stimulated Lrp2-dependent uptake of ApoM-GFP. Dapa attenuated vascular leak induced by LPS in an ApoM-dependent manner.

**Conclusions:**

SGLT2i maintains Lrp2 levels, preserving ApoM and promoting endothelial barrier integrity in acute inflammation, indicating a novel protective mechanism of SGLT2i through ApoM preservation.

Dapagliflozin (Dapa) is a selective sodium-glucose cotransporter 2 inhibitor (SGLT2i), which has well-described clinical benefits in type 2 diabetes mellitus (T2DM), heart failure (HF), and chronic kidney disease.^1^, ^2^, ^3^, ^4^, ^5^ Interestingly, a recent post hoc analysis of Dapa in patients with chronic kidney disease demonstrated reduced infectious death in patients treated with SGLT2i,^6^ and another post hoc analysis of the empagliflozin in HF with preserved ejection fraction trial demonstrated reduced risk of lower respiratory tract infection, findings that are concordant with preclinical studies suggesting that SGLT2i can improve survival in lipopolysaccharide (LPS) murine sepsis models.^7^ Although in patients with COVID-19 and sepsis, SGLT2i has not been shown to significantly reduce all-cause mortality,^8^, ^9^, ^10^, ^11^ wide confidence intervals were observed in these trials that could not exclude benefits (or harms) of these drugs. Further, real-world retrospective data also suggests that SGLT2i may reduce infectious death and acute kidney injury in those already taking these drugs.^12^, ^13^, ^14^, ^15^ However, as in HF and kidney disease, the downstream mechanism by which SGLT2i may lead to these improved outcomes remains enigmatic, particularly with respect to the integrative physiology.

Apolipoprotein M (ApoM) is an apolipoprotein associated with high-density lipoprotein (HDL) that is secreted from hepatocytes and serves as a chaperone for sphingosine-1-phosphate (S1P).^3^^,^^16^, ^17^, ^18^, ^19^, ^20^, ^21^, ^22^ The ApoM/S1P axis is a critical regulator of vascular inflammation and endothelial protection,^3^^,^^16^, ^17^, ^18^, ^19^^,^^21^, ^22^, ^23^, ^24^ and levels of ApoM/S1P have been inversely associated with outcomes in multiple inflammatory conditions including HF,^25^ T2DM,^26^ COVID-19,^27^ and sepsis.^18^^,^^28^, ^29^, ^30^, ^31^ Given that there is an overlap between these associations and the clinical benefits of SGLT2i, notably in T2DM and HF,^32^ we hypothesized that Dapa preserves ApoM levels to maintain vascular integrity in the setting of acute inflammation. We tested this hypothesis in mice using an LPS model, as well as in leveraged patient samples from the Multicenter, Adaptive, Randomized Controlled Platform Trial of the Safety and Efficacy of Antithrombotic Strategies in Hospitalized Adults with COVID-19 (ACTIV-4a) trial to test whether SGLT2i randomization was associated with preserved ApoM vs standard of care (SOC).

## Methods

### Reagents

Dapa was obtained from AstraZeneca (AZ13219875-003), diluted in dimethyl sulfoxide (DMSO) to 100 mg/mL and further diluted in sterile filtered water to achieve the desired concentration. Lipopolysaccharide (LPS) was purchased from Sigma Aldrich (#L2630) and dissolved in 1X phosphate buffered saline, which was used in animal experiments. VPC23019 was purchased from Tocris (#4195) and dissolved in acidified DMSO (5% 1N HCl in DMSO) to 5 mM. For animal experiments, VPC23109 was diluted into sterile normal saline and injected at 0.75 mg/kg. For cell experiments, we used a potent low-density lipoprotein receptor-related protein 2 (LRP2) inhibitor, stable receptor-related protein (RAP),^33^ which was purchased from Kerafast (#EMD007) and prepared to a working solution of 1 μM.

### Murine studies

Mice were maintained on a 12:12-hour light-dark schedule in a temperature-controlled specific pathogen-free facility. Diet-induced obesity (DIO) mice (16-week-old males) were purchased from Jackson Laboratory (C57/BL6J DIO #380050) and maintained on a 60% kCal high-fat, high-calorie diet (Research Diets, D12492). Male mice were gavaged with either Dapa (1.25 mg/kg) or vehicle (Veh) for 4 days prior to LPS injection (10 mg/kg) at 8 pm on day 4. *Apom*^*TG*^ and *Apom*^*KO*^ were previously described.^17^^,^^22^ *Ly6g-cre LoxP-STOP-tdtomato* (Catchup mice) were previously described.^34^ We generated a tamoxifen-inducible proximal tubule-specific *Lrp2*^KO^ by crossing LRP2lox/lox to Slc34a1-CreERT2 mice, previously described.^35^^,^^36^ Mice received tamoxifen by oral gavage, 3 mg every other day for 3 days at 12 weeks of age, and were used for experiments 4 weeks later. Glucose was measured using a Glucocard Vital glucometer (Arkray USA). Creatinine and albumin were measured using the Liasys 330 (AMS Diagnostics). White blood cells and neutrophils were measured using a Hemavet 950 (Drew Scientific).

### Human studies

This study is a post hoc analysis of ApoM measurements from biospecimens collected from a randomized trial completed within the ACTIV-4 platform (NCT04505774), a randomized, shared-placebo platform trial evaluating multiple therapies in patients hospitalized for COVID-19. ACTIV-4a was an international, multicenter, open-label, Bayesian, adaptive randomized platform trial funded by the National Heart, Lung, and Blood Institute (NHLBI). The trial conduct was overseen by a joint clinical coordinating center at the New York University Grossman School of Medicine (Study Chair's office), the University of Pittsburgh School of Medicine (Study Co-Chair's office), Brigham and Women's Hospital and Saint Luke's Mid America Heart Institute, and a data coordinating center at the University of Pittsburgh. Patients were enrolled across several clinical trial networks. Local and/or central institutional review board approval was provided at each site. Informed consent was obtained at the time of enrollment for all patients. This platform trial tested several therapeutic domains (sequentially and concurrently) added to standard of care, including therapeutic-dose heparins, P2Y12 inhibition, P-selectin inhibition with crizanlizumab, and SGLT2 inhibition. Patients who met the inclusion and exclusion criteria were randomly assigned to a locally available SGLT2 inhibitor at each site (Dapa, empagliflozin, canagliflozin, or ertugliflozin at a dose of 10 mg, 10 mg, 100 mg, and 5 mg, respectively; administered orally, once daily for 30 days) in addition to standard of care, or standard of care alone, in an open-label 1:1 ratio. Biospecimens of plasma from ACTIV-4a participants were obtained from the ACTIV Biorepository located at the Laboratory for Clinical Biochemistry Research at the University of Vermont and shipped on dry ice to investigators at the University of Copenhagen for measurement of ApoM by gold-standard enzyme-linked immunosorbent assay (ELISA). Data from ACTIV-4a have been uploaded to BioMed Central.

### Histologic analyses

For immunohistochemistry and tissue histology, kidney tissues were embedded immediately in cryomolds with optimal cutting temperature embedding medium and frozen on dry ice. Immunohistochemical staining was performed with mouse anti-LRP2 (Abcam, ab76969, 1:200). Secondary antibodies were AF488 donkey antirabbit (Invitrogen A21206, 1:200). Confocal images of fluorescent sections were obtained with a Zeiss Axio Imager M2 equipped with a Zeiss LSM 700 laser and a Fujitsu processor. Images were saved as Carl Zeiss Image files using Zen software (Black edition). Image analysis was performed using Visiomorph (VisioPharm). Quantification of mean fluorescence intensity was performed on 9 sections per mouse by NIH ImageJ software.

### Cell experiments

To produce ApoM-GFP, HepG2 cells were grown in a 6-well dish with Dulbecco's modified eagle medium and transfected using lipofectamine 3,000 with a cytomegalovirus-driven construct containing the human *APOM* sequence and GFP. The expression of green fluorescent protein labeled human ApoM (ApoM-GFP) was verified by Western blot analysis, microscopy, and quantitative real-time polymerase chain reaction (qRT-PCR). Cell medium was harvested after 48 hours and concentrated using Amicon Ultra 30K spin columns. The concentration of ApoM-GFP in collected cell medium was measured by EnSpire. For experimental setups, HK2 cells were grown on 8-chamber slides for 24 hours, and the cells were thereafter exposed to Dapa (6 nM) or vehicle for 16 hours, followed by addition of LPS (0.1 μg/mL) or saline, as well as ApoM-GFP. The LPS stimulation and addition of ApoM-GFP were done for 2-6-16 hours with 8000-16000-24000-48000 arb units. To test the role of LRP2, HK2 cells were grown on 8-chamber slides for 24 hours, and the cells were thereafter exposed to Dapa (6 nM) or vehicle for 16 hours, followed by addition of RAP (RAP, Kerafast, 1 μM) 1 hour prior to LPS (0.1 μg/mL) or saline, as well as ApoM-GFP (24,000 arb units). The cells were mounted with Fluoroshield. Images were obtained using a Zeiss Axio Scan.Z1. Quantification of mean fluorescence intensity was performed by ImageJ software.

### Quantitative real-time polymerase chain reaction analysis

qPCR was performed as described.^37^ Briefly, total RNA in kidney and liver tissues were extracted using RNeasy Mini kit (Qiagen, #74104), and the first-strand cDNA was prepared using the iScriptTM cDNA synthesis kit (Bio-Rad, #1708890). qPCR analysis was performed with SYBR Green Master Mix (Bio-Rad, #1725121) on QuantStudio 3 Real-Time PCR system (Applied Biosystems, A28136) to determine relative mRNA levels. Mice sequences for qRT-PCR primers are shown in Supplemental Table 1.

### Western blot analysis

Western blot was performed on plasma and tissue samples from mice as previously described.^38^ Primary antibodies employed were as follows: mouse ApoM (LS Bio, C158166, diluted 1:1000), low-density lipoprotein receptor (LDLR) (Abcam, ab52818, diluted 1:500), and β-actin (Sigma-Aldrich, A2066, diluted 1:4,000). The band intensity was measured and analyzed with ImageJ software.

### ApoM ELISA methods

Measurements of mouse ApoM were performed using a competitive ELISA described previously.^39^ In short, samples were prepared by diluting mouse plasma 1:3,500 in tris-buffered saline (TBS), which contains an in-house produced polyclonal rabbit anti-apoM antibody with 1:600 dilution and incubating for approximately 16 hours. Meanwhile, a high-binding Costar 96-well ELISA plate (cat no. 3590) was coated with 20 μg/mL of wild-type mouse HDL fraction (obtained by ultracentrifugation of plasma) in TBS. The plate was subsequently washed 5 times and quenched with TBS + 1% bovine serum albumin for 2 hours before samples were added for 1 hour. After washing, a horseradish peroxidase-conjugated goat antirabbit IgG polyclonal antibody (Dako, cat. no. P0448) was added at 1:2000 for 1 hour. The bound antibody was detected with o-phenylenediamine and hydrogen peroxide in H_2_O, and absorbance was read at 492 nm. A standard curve generated from a pool of wild-type mouse plasma was used to calculate relative concentrations.

### Intravital microscopy

*Ly6G-Cre LoxP-STOP-TdTomato* mice on a chow diet were treated with vehicle or VPC23019 (0.75 mg/kg body weight IP daily) and gavaged with vehicle or Dapa (1.25 mg/kg daily) for 4 days before IP injection of LPS (7.5 mg/kg). Twenty-four hours after LPS challenge, intravital microscopy was performed as we described.^40^ Fluorescein isothiocyanate (FITC)-conjugated dextran (1 mg/g) was infused into a jugular venous cannulus. Neutrophil recruitment and vascular leak were monitored in an area of 0.02 mm^2^ (number/field/5 minutes) in the inflamed cremaster venules with a diameter of 25 to 40 μm. The number of neutrophils that visibly rolled over the inflamed endothelium over 5 minutes was counted by an observer blinded to the treatment groups and unfamiliar with the study hypotheses. Adherent neutrophils were defined as neutrophils that were stationary for more than 30 seconds or crawled on the inflamed endothelium but did not roll over. Vascular leak was calculated by subtraction of the median fluorescence intensities of FITC-conjugated dextran inside of blood vessels from those of FITC-conjugated dextran in the field of view. The median extravascular FITC intensity per unit time (across the first 5 randomly selected vessels) and the per-vessel area under the curve were calculated. In independent experiments, wild type littermate controls, *Apom*^*KO*^, and *Apom*^*TG*^ (along with their respective littermates) mice on a chow diet were gavaged with vehicle or Dapa for 4 days prior to LPS injection. Vascular leak and neutrophil recruitment were monitored by injection of FITC-conjugated dextran and Alexa Fluor 647-conjugated anti-Ly-6G antibodies (0.1 μg/g), respectively. Fluorescence and bright-field images were recorded using a Zeiss Axio examiner Z1 microscope system with a Yokogawa confocal spinning disk (CSU-W1) equipped with 4-stack laser system (405 nm, 488 nm, 561 nm, and 637 nm wavelengths). Images were collected with a high-speed, high-resolution camera (2,304 × 2,304 pixel format, ORCA-Fusion BT sCMOS, Hamamatsu). Data were analyzed using SlideBook, version 6.0 (Intelligent Imaging Innovations). Each curve is the median extravascular FITC-Dextran intensity across the quantified vessels (n = 5 vessels per mouse, 3-5 mice per group).

### Statistical analyses

We performed a power analysis to determine the sample size needed to detect a 50% difference in ApoM between the LPS and Dapa + LPS groups with 80% power and an alpha level of 0.05. The analysis revealed that approximately 11 subjects per group are required to achieve these parameters. Data were considered non-normally distributed if either the Shapiro-Wilk or Kolmogorov-Smirnov tests were statistically significant (*P* < 0.05). If the data were normally distributed, we used the 2-tailed Student's *t*-test for 2 group comparisons. For comparison among multiple groups, we used a parametric analysis of variance with Sidak's post hoc testing for multiple comparison corrections as specified in each figure legend. If the data were not normally distributed, Kruskal-Wallis testing (followed by Dunn test) was used instead of analysis of variance. Differences in baseline characteristics between patients who were and those who were not treated with an SGLT2 inhibitor were compared by Student's *t*-test for continuous variables and by the chi-square test or Fisher exact test for categorical variables. In all the analyses, a value of *P* < 0.05 was considered statistically significant. Data are presented as mean ± SD or median ± IQR and analyzed by GraphPad Prism 10.4.0.

### Study approval

All murine studies were approved by the Institutional Animal Care and Usage Committee at Washington University in St. Louis and were performed following the Guide for the Care and Use of Laboratory Animals. Animal procedures were carried out in accordance with the Washington University School of Medicine Animal Studies Committee, which approved the protocols.

## Results

### Dapagliflozin and circulating protein levels of ApoM in LPS-treated mice

**Figure 1:**
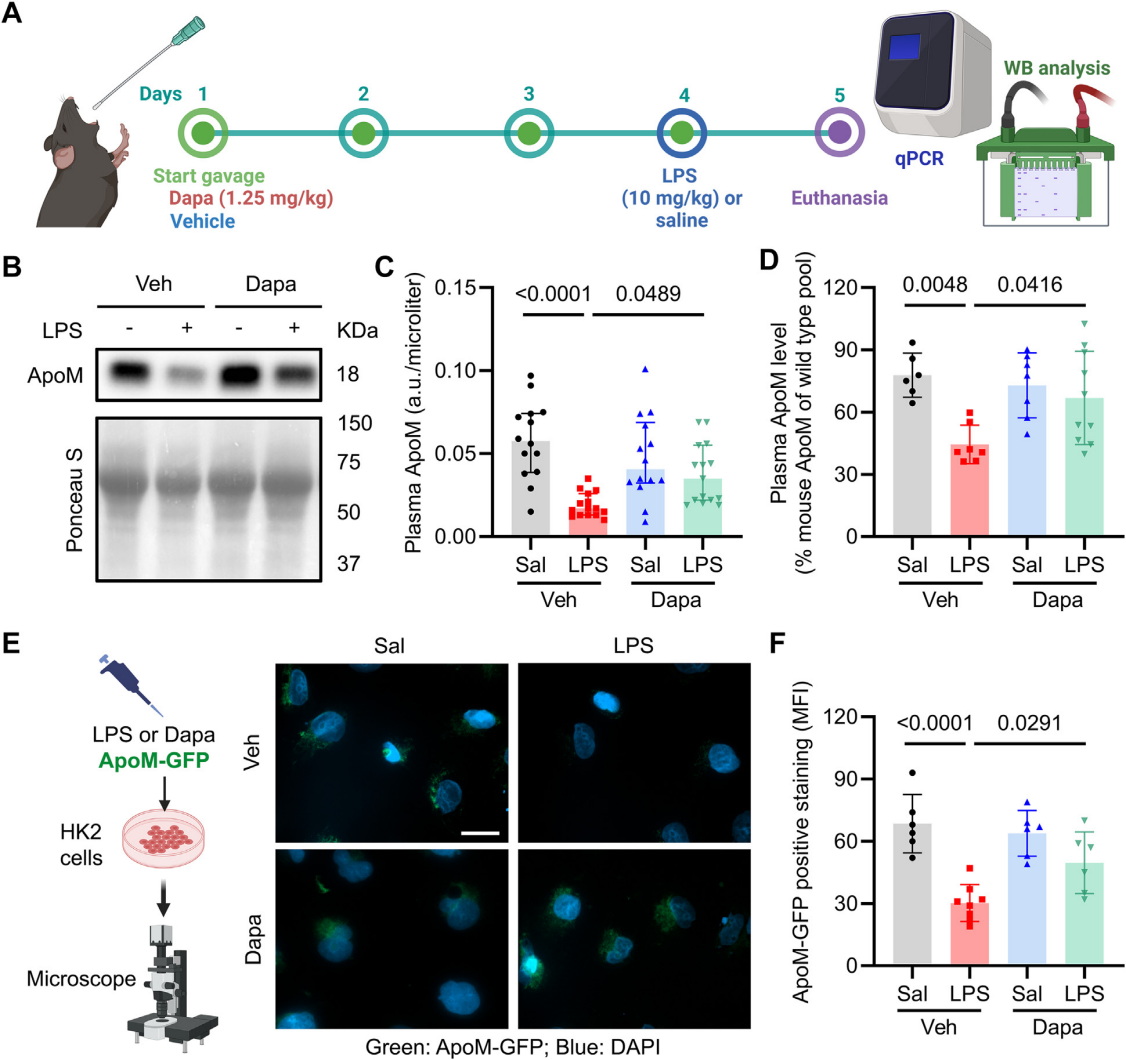
**Dapagliflozin Preserves Circulating Apolipoprotein M in LPS-Treated Mice** (A) Diet-induced obesity mice were randomized to vehicle or Dapa (1.25 mg/kg gavage daily for 4 days) before saline or LPS (10 mg/kg IP) and euthanized the next day. (B) Representative images of immunoblots for murine ApoM from plasma with quantification in (C) (n = 14-15/group); (D) plasma ApoM level by ELISA (n = 6-10/group). (E) Representative fluorescence images were obtained from HK2 cells, which were grown on 8-chamber slides for 24 hours, thereafter, exposed to Dapa (6 nM) or vehicle for 16 hours, followed by addition of saline or LPS (0.1 μg/mL) as well as ApoM-GFP (24,000 arb units) for further 6 hours (scale bar = 20 μm). (F) Quantification of mean fluorescence intensity in (H) (n = 6-8/group). All data are presented as mean ± SD or median ± IQR. Each dot represents 1 mouse. Two-way ANOVA with Sidak's correction for multiple comparisons in panels (C, D, and F). ANOVA = analysis of variance; ApoM = apolipoprotein M; ApoM-GFP = green fluorescent protein labeled human ApoM; DAPI = 4’,6-diamidino-2-phenylindole; ELISA = enzyme-linked immunosorbent assay; LPS = lipopolysaccharide.

To test our hypothesis that Dapa preserves circulating ApoM in acute inflammation, we utilized a model of DIO generated by feeding mice 60% high-fat, high-calorie diet from 6 to 16 weeks of age. We performed studies in DIO mice because it is a well-described model of prediabetes, impaired glucose tolerance, and obesity,^41^^,^^42^ pathologies known to reduce ApoM.^43^^,^^44^ DIO mice underwent oral gavage with vehicle or Dapa (1.25 mg/kg daily) prior to saline or LPS injection (10 mg/kg IP) on day 4, followed by in vivo phenotyping and euthanasia on day 5 (Figure 1A). LPS administration-induced hypothermia, weight loss, and transient hypoglycemia, none of which was influenced by Dapa (Supplemental Figure 1).

In DIO mice, Dapa pretreatment significantly attenuated LPS-induced decreases in circulating ApoM (Figure 1B-D), as shown by Western blot and ELISA.^39^^,^^45^ In contrast, Dapa did not have a significant effect on serum albumin concentrations (Supplemental Figure 2). The relationship between SGLT2i and ApoM uptake was further evaluated in vitro using human proximal tubular (HK2) cells treated with either LPS or LPS and Dapa. The uptake of ApoM-GFP was quantified, and the results support that Dapa preserved the uptake of ApoM-GFP in renal proximal tubular cells under inflammatory conditions (Figures 1E and 1F, Supplemental Figure 3).

### Dapagliflozin and circulating protein levels of ApoM in humans with COVID-19

**Figure 2:**
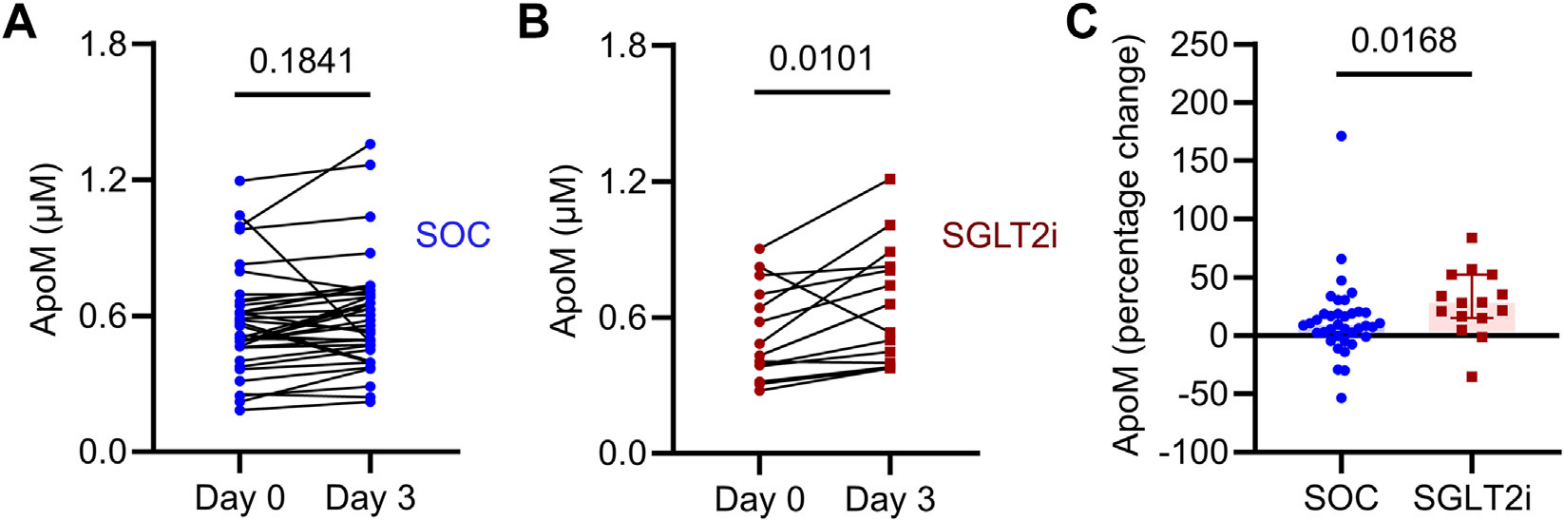
**Dapagliflozin Preserves Circulating Apolipoprotein M in Human With COVID-19** (A and B) Circulating ApoM in patients randomized to SOC (n = 37) vs SGLT2i (n = 15) who had banked plasma available at day 0 and day 3. (C) Percentage change in ApoM on day 3 vs day 0 in patients randomized to standard of care vs SGLT2i. All data are presented as mean ± SD or median ± IQR. Each dot represents 1 person. Student's *t*-test for in panels (A and B), and Mann-Whitney test in C. ApoM = apolipoprotein M; SGLT2i = sodium-glucose co-transporter 2 inhibitor; SOC = standard of care.

To determine the clinical relevance of this observation, we utilized banked samples from ACTIV-4a, an adaptive platform trial that randomized patients to SOC vs SGLT2i (among other therapies) in COVID-19. Reduced ApoM has been previously described as a negative prognostic indicator in patients with COVID-19.^46^^,^^47^ Circulating ApoM was measured by ELISA, as previously described.^25^ ApoM levels were generally low, consistent with prior observations of acutely and persistently reduced ApoM in patients hospitalized with COVID-19.^25^^,^^46^, ^47^, ^48^ Characteristics of patients randomized to either SOC alone or SGLT2i alone, with available biorepository samples at day 0 are shown in Table 1. In ACTIV-4a, mean baseline ApoM was 0.547 ± 0.216. Baseline ApoM was associated with reduced odds of the composite endpoint of organ support or mortality (OR 0.673, 95% CI 0.515-0.880, *P* = 0.004, per 0.1 μM ApoM), while randomization to SGLT2i was associated with increased percentage change in ApoM vs SOC on day 3 (Figure 2). These findings highlight the potential translational relevance of our findings that SGLT2i can preserve ApoM levels.

**Table 1:** Baseline Characteristics by SGLT2i Use

	SOC (n = 37)	SOC+SGLT2i (n = 15)	*P* Value
ApoM (μM)	0.5 [0.5, 0.6]	0.5 [0.4, 0.7]	0.42
Age, y^a^	63.3 ± 11.8	76.0 ± 6.0	0.036
Age >65 y	26 (70.3%)	13 (86.7%)	0.22
Female	21 (56.8%)	7 (46.7%)	0.51
Race			0.42
Asian	3 (8.1%)	0 (0.0%)	
Black or African American	6 (16.2%)	5 (33.3%)	
White	25 (67.6%)	9 (60.0%)	
Unknown	3 (8.1%)	1 (6.7%)	
Hispanic ethnic	32 (91.4%)	14 (93.3%)	0.82
BMI kg/m^2^	32.7 ± 10.4	29.5 ± 11.8	0.35
Heart rate/min^a^	74.9 ± 15.4	84.7 ± 14.7	0.042
Systolic blood pressure (mm Hg)	130.8 ± 22.6	134.1 ± 15.8	0.61
Medical history			
Hypertension	26 (70.3%)	14 (93.3%)	0.07
Heart failure	8 (21.6%)	4 (26.7%)	0.70
Coronary artery disease	13 (35.1%)	4 (26.7%)	0.56
Peripheral arterial disease	3 (8.1%)	0 (0.0%)	0.26
Cerebrovascular disease (stroke or TIA)	4 (10.8%)	3 (20.0%)	0.38
Cardiac arrhythmia	8 (21.6%)	0 (0.0%)	0.05
Cerebrovascular disease (stroke or TIA)	4 (10.8%)	3 (20.0%)	0.38
Chronic kidney disease	12 (32.4%)	4 (26.7%)	0.68
Liver disease	2 (5.4%)	0 (0.0%)	0.36
Asthma	3 (8.1%)	0 (0.0%)	0.26
COPD	9 (24.3%)	4 (26.7%)	0.86
Smoking status			0.20
Current	4 (11.4%)	0 (0.0%)	
Former	20 (57.1%)	7 (46.7%)	
Never	11 (31.4%)	8 (53.3%)	
Baseline treatment			
Steroids	26 (70.3%)	9 (60.0%)	0.47
Antiplatelets	12 (32.4%)	7 (46.7%)	0.33
Anticoagulant therapies	30 (81.1%)	12 (80.0%)	0.93
Remdesivir	30 (83.3%)	11 (73.3%)	0.41
IL-6 inhibitors	0 (0.0%)	0 (0.0%)	
Baricitinib	4 (10.8%)	0 (0.0%)	0.19
ACEI	6 (16.2%)	5 (33.3%)	0.17
ARB	7 (18.9%)	2 (13.3%)	0.63
Aldosterone antagonist	0 (0.0%)	1 (6.7%)	0.11
Beta-blocker	18 (48.6%)	7 (46.7%)	0.90
Statins	19 (51.4%)	9 (60.0%)	0.57
Organ support			
None, nasal cannula, venturi mask	31 (83.8%)	13 (86.7%)	0.79
High-flow nasal cannula (>20 L/min)	6 (16.2%)	2 (13.3%)	0.79
Noninvasive ventilation	1 (2.7%)	1 (6.7%)	0.50
Invasive mechanical ventilation	1 (2.7%)	0 (0.0%)	0.52
Vasopressors or inotropes	0 (0.0%)	0 (0.0%)	

ACEI = angiotensin-converting enzyme inhibitor; ApoM = apolipoprotein M; ARB = angiotensin receptor blocker; BMI = body mass index; COPD = chronic obstructive pulmonary disease; IL = interleukin; SGLT2i = sodium-glucose co-transporter 2 inhibitor; SOC = standard of care; TIA = transient ischemic attack.

a *P* < 0.05 for the difference between groups.

### Dapagliflozin increases circulating ApoM via the Lrp2 scavenger receptor

**Figure 3:**
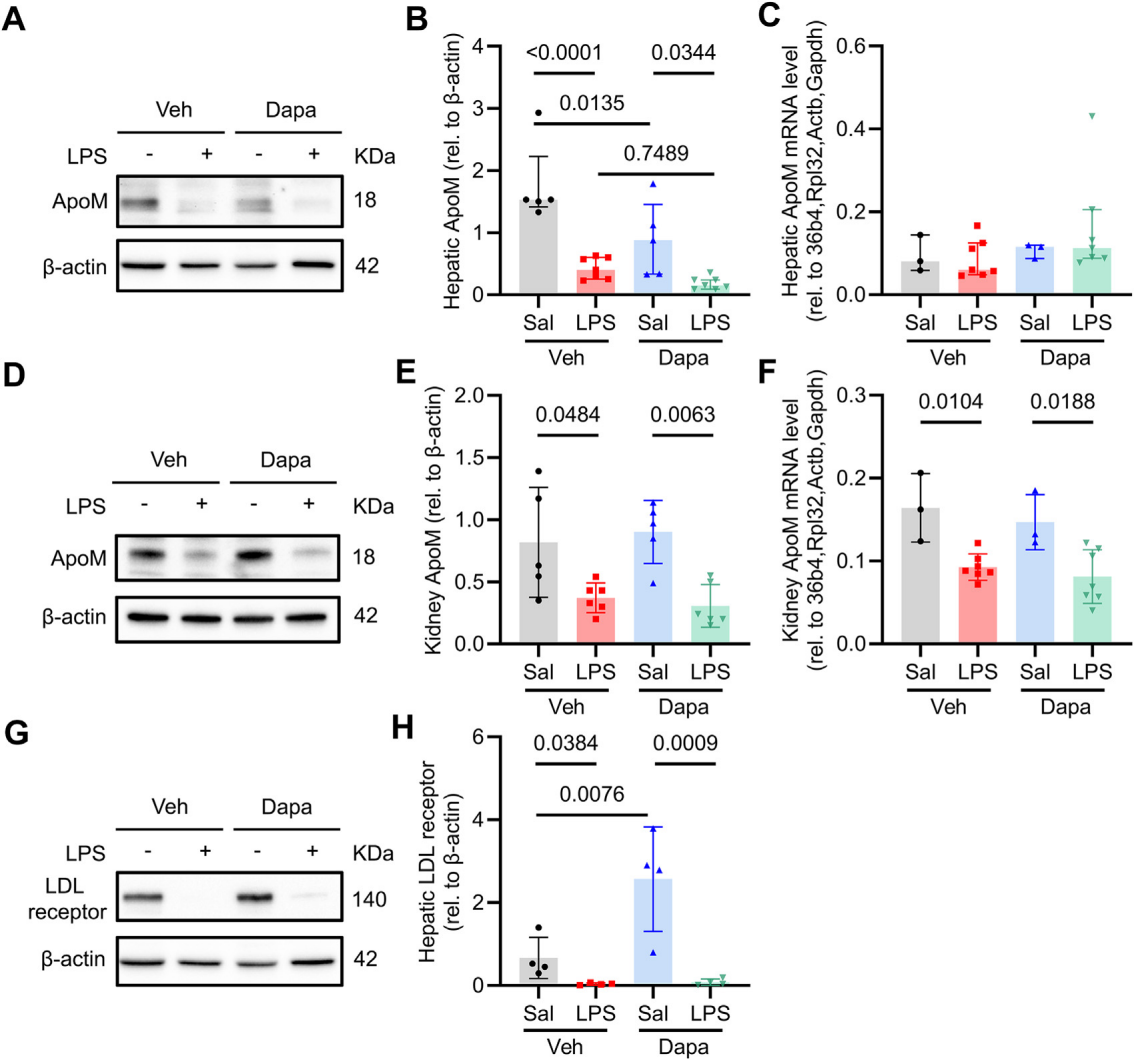
**Lipopolysaccharide Reduces Apolipoprotein M Protein and mRNA Abundance in Liver and Kidney** Diet-induced obesity mice were randomized to vehicle or Dapa (1.25 mg/kg gavaged daily for 4 days) before saline or LPS (10 mg/kg IP) and euthanized the next day. (A) Representative images of immunoblots for ApoM from hepatic protein isolates with quantification in (B) and hepatic ApoM mRNA abundance in (C) (n = 3-7/group); (D) representative images of immunoblots for kidney ApoM with quantification in (E) and renal ApoM mRNA abundance in (F) (n = 3-7/group). (G) Representative images of immunoblots for hepatic LDLR and quantification in (H) (n = 4/group). β-Actin serves as loading control for (A, B, D, E, G, and H). All data are presented as mean ± SD or median ± IQR. Each dot represents 1 mouse. Two-way ANOVA with Sidak's correction for multiple comparisons in panels (B, C, E, F, and H). ANOVA = analysis of variance; ApoM = apolipoprotein M; LDLR = low-density lipoprotein receptor; LPS = lipopolysaccharide.

Next, we investigated how Dapa might attenuate LPS-induced reductions in ApoM. The liver, and to a lesser extent the kidney, are the main sites of ApoM production.^28^ In our model, Dapa reduced serum creatinine (Supplemental Figure 4), consistent with prior data that SGLT2i attenuated renal injury in LPS-treated mice.^7^ We found that while LPS reduces both hepatic and renal ApoM protein abundance, as well as kidney mRNA abundance, none of these were affected by Dapa (Figures 3A to 3F). Since we did not find considerable changes in ApoM mRNA and protein abundance in either the liver or the kidney, we sought to examine other potential mechanisms by which Dapa might preserve circulating ApoM.

Hepatic ApoM clearance is thought to be dependent on LDLR uptake;^49^ however, LPS suppressed LDLR equally in both vehicle- and Dapa-treated mice (Figures 3G and 3H), suggesting that hepatic ApoM clearance was highly unlikely to be responsible for the observed increase in circulating ApoM in this context.

**Figure 4:**
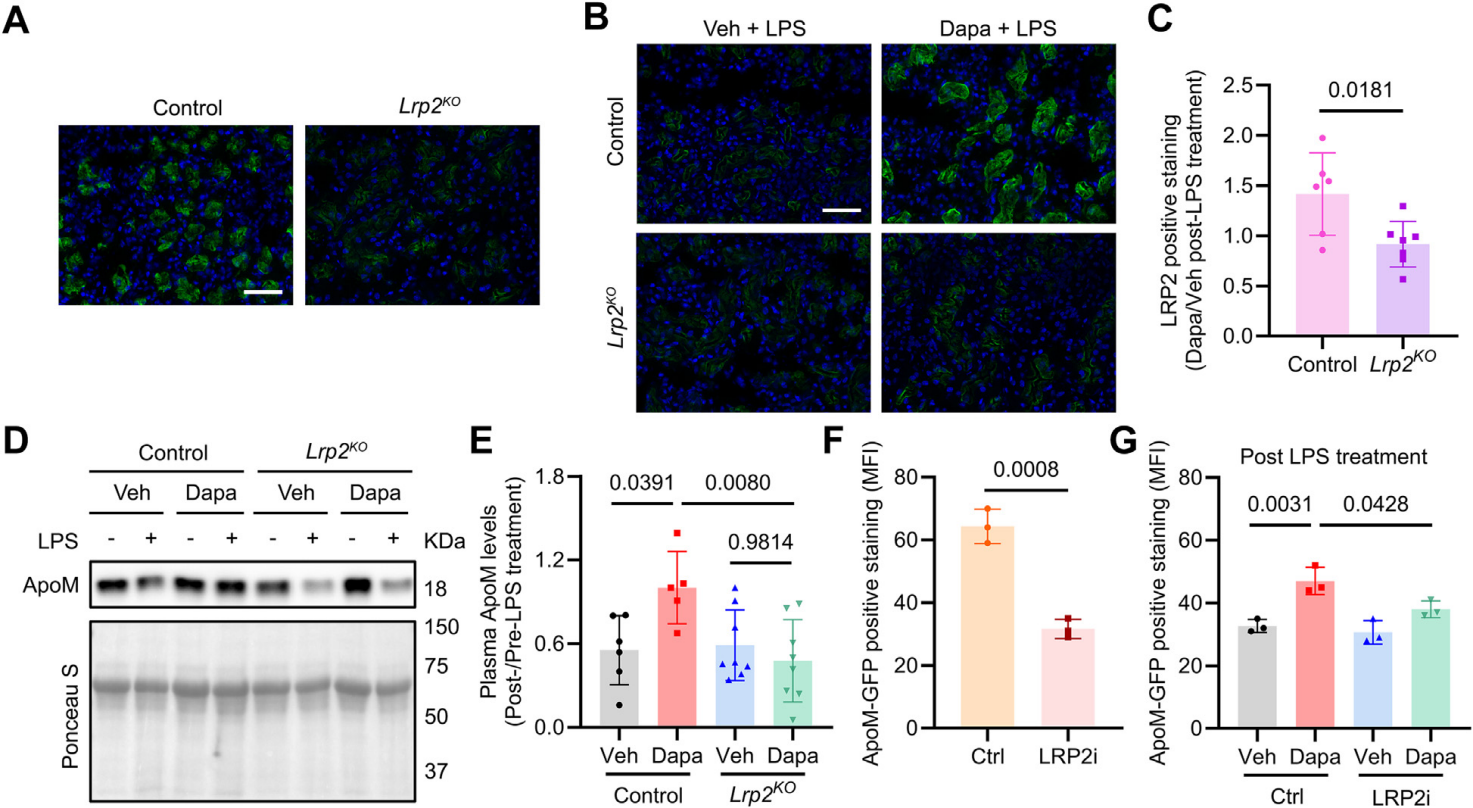
**Proximal Tubule-Specific Knockout or Inhibition of Lrp2 Attenuates the Effects of Dapagliflozin on ApoM Maintenance** (A) Representative immunofluorescence staining images for Lrp2 (green) and DAPI (blue) in kidney frozen sections from *Lrp2*^*KO*^ and littermate controls (scale bar = 50 μm). (B) Representative immunofluorescence staining images for Lrp2 (green) and DAPI (blue) in kidney frozen sections from *Lrp2*^*KO*^ and littermate controls randomized to vehicle or Dapa treatment (1.25 mg/kg daily for 4 days) and treated with LPS (10 mg/kg IP), with (C) quantification of LRP2 MFI, shown as fold change of Dapa to vehicle (n = 6-7/group; scale bar = 50 μm). (D) Representative images of immunoblots for ApoM from paired plasma with quantification in (E) from *Lrp2*^*KO*^ and littermate control mice randomized to vehicle or Dapa (1.25 mg/kg gavage daily for 4 days) before saline or LPS (10 mg/kg IP) and euthanized the next day (n = 5-8/group). (F) MFI of ApoM-GFP in HK2 cells which were grown on 8-chamber slides for 24 hours and then treated with 1 μM LRP inhibitor for 1 hour prior to ApoM-GFP (24,000 arb units) addition (n = 3). (G) MFI of ApoM-GFP in above HK2 cells setting with additional treatment including exposure to Dapa (6 nM) or vehicle for 16 hours and followed by addition of LPS (0.1 μg/mL) or saline prior to ApoM-GFP addition (n = 3/group). All data are presented as mean ± SD or median ± IQR. Each dot represents 1 mouse or 1 biological replicate (individual wells). Student's *t*-test in panels (C and F). Two-way ANOVA with Sidak's correction for multiple comparisons in panels (E and G). ANOVA = analysis of variance; ApoM = apolipoprotein M; DAPI = 4’,6-diamidino-2-phenylindole; LPS = lipopolysaccharide; LRP2, low-density lipoprotein receptor-related protein 2; MFI = mean fluorescence intensity.

Alternatively, we hypothesized that Dapa regulates ApoM reabsorption, which occurs in the renal proximal tubule by the protein receptor Lrp2.^50^ Megalin or LDLR-related protein 2 (Lrp2), a 600 kDa protein receptor and member of the LDLR family, is downregulated by high glucose and LPS,^51^, ^52^, ^53^ and critical for renal reabsorption of ApoM under conditions of kidney injury.^54^ Since the proximal tubule is also the site of SGLT2i/Dapa action, we hypothesized that Dapa may prevent LPS-induced reduction in Lrp2 levels. Blinded quantification of integrated fluorescent intensity from murine kidney sections stained with Lrp2 antibody showed that LPS-treated mice that received Dapa had increased Lrp2 immunofluorescence compared to controls (Supplemental Figure 5). To validate our Lrp2 immunofluorescence and establish a mechanistic role for Lrp2 downstream of SGLT2i, we modeled proximal tubule-specific Lrp2 knockout mice (*Lrp2*^KO^), generated by crossing Slc34a1-CreERt2 mice to *Lrp2*^flox^ mice, as previously described.^35^^,^^36^ After tamoxifen oral gavage (3 mg × 3 days), we validated *Lrp2* knockout by immunofluorescence. *Lrp2*^KO^ mice showed lower levels of Lrp2 immunofluorescence from kidney sections stained with Lrp2 antibody compared with littermate controls (*Lrp2*^flox^) (Figure 4A). Given that Dapa preserves circulating ApoM and prevents LPS-induced reductions in Lrp2, we next sought to test whether knocking out Lrp2 in proximal tubules attenuates the effects of Dapa on circulating ApoM. To determine if Lrp2 is required for the effects of Dapa on ApoM levels, *Lrp2*^KO^ and littermate control mice were randomized to vehicle vs Dapa (1.25 mg/kg gavage daily for 4 days) and treated with saline vs LPS (10 mg/kg IP) the day after the last dose of Dapa. While control mice treated with Dapa preserved Lrp2 levels after LPS treatment, Dapa did not rescue Lrp2 levels in *Lrp2*^KO^ mice (Figures 4B and 4C). In addition, Dapa did not show preserved circulating ApoM levels in *Lrp2*^KO^ mice treated with LPS (Figures 4D and 4E). As a further test of the hypothesis that Dapa rescues circulating ApoM by preserving Lrp2 levels in the proximal tubule, we performed in vitro experiments with an HK2 cell line and an Lrp2 inhibitor (LRP2i), RAP.^33^ Mean fluorescent intensity (MFI) of ApoM-GFP was significantly lower in cells treated with 1 μM Lrp2 inhibitor for 1 hour prior to ApoM-GFP addition (Figure 4F). Subsequently, HK2 cells treated with the LRP2i vs control were treated with Dapa vs vehicle for 16 hours, followed by the addition of LPS (0.1 μg/mL) or saline prior to ApoM-GFP addition. While Dapa treatment showed an increase in MFI of ApoM-GFP compared to vehicle controls, LRP2i attenuated Dapa-induced increases in MFI of ApoM-GFP (Figure 4G).

### Dapagliflozin attenuates vascular leak and neutrophil transendothelial migration via ApoM/S1P

**Figure 5:**
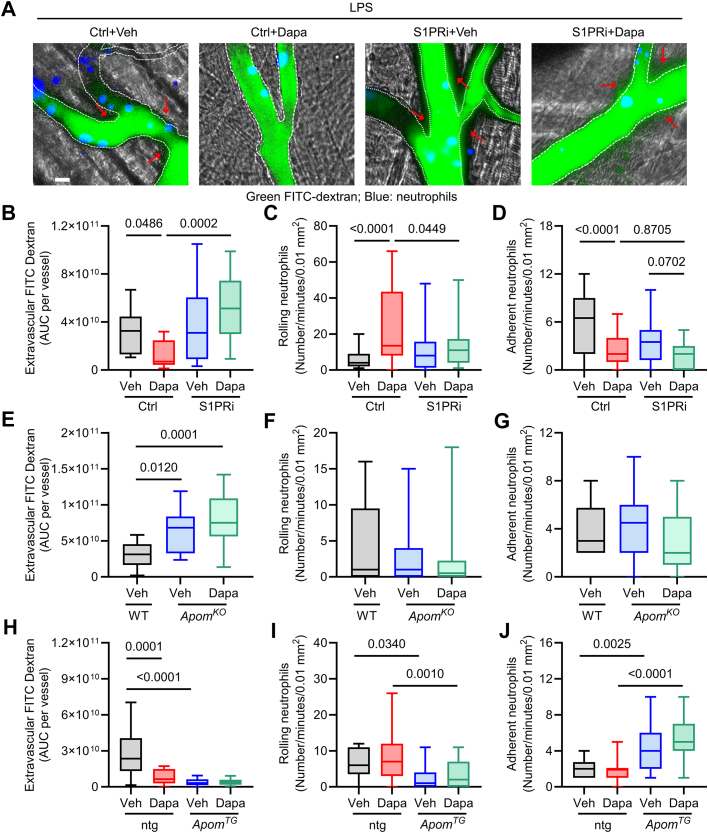
**Dapagliflozin Attenuates LPS-Induced Endothelial Leak in an S1P and ApoM-Dependent Manner** *Ly6G-*Cre-TdTomato mice on chow diet were treated with control or S1PRi (0.75 mg/kg IP daily) and gavaged with vehicle or Dapa (1.25 mg/kg daily for 4 days) before LPS (7.5 mg/kg IP). Intravital microscopy was performed. (A) Representative images of cremaster venules, FITC-Dextran (green), neutrophils (pseudo color blue), white dotted lines indicate vessel border, and red arrows highlighting areas of extravascular FITC-Dextran (scale bar = 10 μm); (B) AUC per vessel of extravascular FITC-Dextran; (C) The numbers of rolling and (D) adherent neutrophils observed in 5 minutes; (E-G) Littermate controls (wild type) gavaged with vehicle for 4 days then treated with LPS compared to *Apom*^*KO*^ mice fed a chow diet and treated with vehicle or dapagliflozin gavage for 4 days prior to LPS injection. Intravital microscopy was performed to assess extravascular FITC-Dextran and rolling, and adherent neutrophils; (H-J) Littermate control nontransgenic mice (ntg) vs *Apom*^*TG*^ mice on chow diet were gavaged with vehicle or Dapa (1.25 mg/kg daily for 4 days) prior to LPS injection and intravital microscopy for assessment of extravascular FITC-Dextran and rolling and adherent neutrophils. All experiments quantified vessels >4 per mouse (n = 3-5 mice per group). All data are presented as min to max in box plot. One way ANOVA with Sidak's correction for multiple comparisons in panels (E-G); Two-way ANOVA with Sidak's correction for multiple comparisons in panels (B-D) and (H-J). ANOVA = analysis of variance; AUC = area under the curve; FITC = fluorescein isothiocyanate; LPS = lipopolysaccharide.

One function of ApoM/S1P is to maintain endothelial barrier integrity and therefore intravascular volume. Previously, SGLT2i have been described to preserve endothelial integrity, although how the actions of SGLT2i are tied to this vascular protective mechanism has remained elusive. We hypothesized that preservation of ApoM/S1P signaling was critical for maintenance of endothelial barrier integrity and intravascular volume by Dapa. To test this, we investigated the effects of Dapa on endothelial leak, as well as neutrophil rolling and adhesion on cremaster venules by intravital microscopy (IVM). We visualized the cremaster venules utilizing confocal IVM in chow-fed *Ly6G-Cre LoxP-STOP-TdTomato* mice,^34^ in which neutrophils express TdTomato. Simultaneous injection of FITC-dextran allows assessment of endothelial leak. To examine whether the beneficial effect of Dapa is derived from S1P signaling, we tested the combined effect of Dapa and an S1P receptor 1 and 3 inhibitor, VPC23019 (S1PRi). Blinded quantification of fluorescent signal outside of the vessel showed that, compared with vehicle, Dapa treatment significantly decreased vascular leak induced by LPS (Figures 5A and 5B). Moreover, treatment with S1PRi blocked the effect of Dapa on FITC-dextran extravasation (Figures 5A and 5B). Pretreatment with Dapa increased the number of rolling neutrophils and decreased the number of adherent neutrophils (Figures 5C and 5D); nonetheless, S1PRi blocked the effects of Dapa on rolling neutrophils (Figure 5C), but not adherent neutrophils (Figure 5D), implying that Dapa in part affects neutrophil recruitment in a S1PR-dependent manner. Importantly, we did not observe any consistent effects of Dapa on S1P receptor expression (Supplemental Figure 6), or significant increases in the number of circulating neutrophils (Supplemental Figure 7). These results indicate that Dapa reduces vascular leak and neutrophil recruitment induced by LPS, an effect dependent on S1PR signaling.

Given that Dapa prevented reductions in circulating ApoM in LPS-treated DIO mice, we next sought to determine whether ApoM mediated the effects of Dapa on endothelial integrity and neutrophil recruitment. To test this, we utilized both ApoM knockout mice (*Apom*^*KO*^), which exhibit a 50% reduction in plasma S1P,^17^ and mice with hepatocyte-specific overexpression of human *APOM* (*Apom*^*TG*^), which results in a 3- to 5-fold increase in plasma ApoM and S1P.^22^ While *Apom*^*KO*^ mice exhibited increased vascular leak compared to littermate controls following LPS, Dapa did not significantly reduce FITC-dextran extravasation in *Apom*^*KO*^ mice (Figure 5E). Compared to littermate controls, *Apom*^*KO*^ mice did not affect neutrophil rolling and adhesion on the inflamed endothelium (Figures 5F and 5G). In contrast, IVM demonstrated that *Apom*^*TG*^ mice exhibit reduced vascular leak (Figure 5H), fewer rolling neutrophils (Figure 5I), and more adherent neutrophils (Figure 5J), but Dapa pretreatment did not significantly alter any of these parameters in *Apom*^*TG*^ mice. These results suggest that Dapa reduces LPS-induced endothelial leak via ApoM-S1P signaling, but that the effect of Dapa on neutrophil recruitment may only be partially dependent on ApoM/S1P.

## Discussion

We have uncovered that treatment with the SGLT2i Dapa preserves circulating ApoM in mice given LPS to maintain vascular integrity, findings that were corroborated by evidence from ACTIV-4a, where patients randomized to SGLT2i had relatively increased ApoM compared to those receiving SOC. We propose a model whereby, in the setting of systemic inflammation, Dapa preserves Lrp2 levels, leading to conservation of circulating ApoM, which in turn attenuates LPS-induced endothelial vascular permeability and inflammation (Central Illustration). The most important finding of our study is that pretreatment with Dapa reduces LPS-induced endothelial leak in an ApoM/S1P-dependent manner, which is of clinical importance in patients experiencing a severe inflammatory condition.

**Central Illustration:**
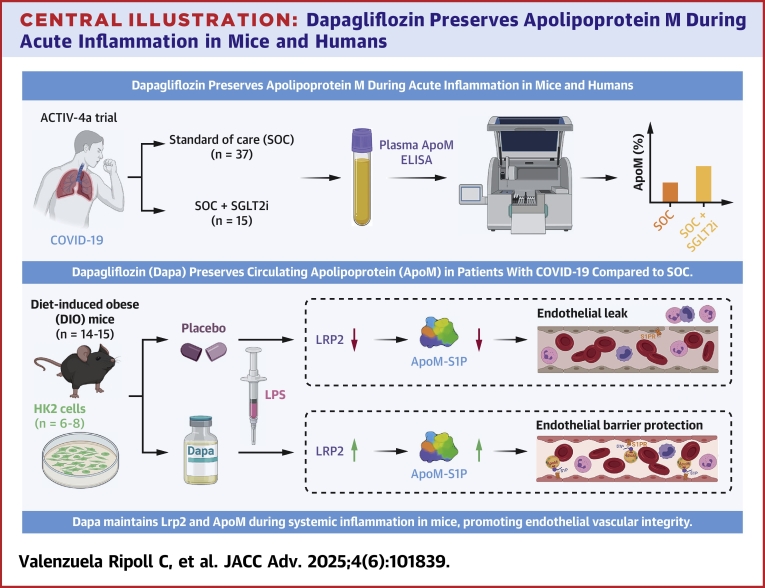
**Dapagliflozin Preserves Apolipoprotein M During Acute Inflammation in Mice and Humans** In humans from the ACTIV-4a trial, randomization to sodium-glucose co-transporter inhibitors increased circulating ApoM levels. In mice, dapagliflozin pretreatment attenuated loss of the scavenger receptor LRP2 and ApoM, defending against lipopolysaccharide-induced disruption of endothelial barrier integrity. ApoM = apolipoprotein M; LRP2 = low-density lipoprotein receptor-related protein 2; SGLT2i = sodium-glucose co-transporter 2 inhibitor; SOC = standard of care.

Our findings have multiple translational implications and link SGLT2i to the ApoM/S1P pathway, which is inversely associated with mortality in COVID-19,^27^^,^^47^ sepsis,^18^^,^^28^, ^29^, ^30^ HF ^25^, and T2DM.^18^^,^^26^^,^^28^, ^29^, ^30^ ApoM also represents an important connection between diabetes and HF with preserved ejection fraction, a condition where SGLT2i recently demonstrated benefits.^55^^,^^56^ Moreover, it is known that ApoM reduces endothelial inflammation and preserves barrier integrity through endothelial S1P signaling.^16^^,^^17^^,^^19^^,^^57^^,^^58^ Clinical studies suggest that SGLT2i acutely reduce excess extravascular volume.^59^ We identify a mechanism by which SGLT2 inhibition with Dapa in the proximal tubule preserves levels of ApoM via increased reuptake mediated by Lrp2 contributing to preserved endothelial barrier integrity. Results from our IVM studies show that extravascular leak was increased in *Apom*^*KO*^ but reduced in *Apom*^*TG*^ mice, with no additional effect of Dapa. Further, pharmacological inhibition of S1P receptors abrogated the effects of Dapa on endothelial barrier maintenance, demonstrating that the ApoM/S1P signaling pathway is necessary and sufficient for the effects of Dapa on endothelial leak.

Our results suggest that Dapa does not upregulate ApoM synthesis in the liver or kidney, the primary producers of ApoM. Rather, Dapa appears to preserve ApoM levels in the setting of an acute inflammatory reaction through reabsorption in the proximal tubule via upregulation of Lrp2. Although a prior study suggested that SGLT2i may reduce Lrp2 protein levels in a chronic type I diabetic mouse model,^60^ our present studies instead utilized both mice fed a high-fat, high-calorie diet and a chow diet. Interestingly, Lrp2 is downregulated both by hyperglycemia and LPS, conditions associated with urinary protein loss. Our finding that Dapa preserves Lrp2 is concordant with the clinical effects of SGLT2i, reducing renal protein loss in patients with type 2 diabetes.^61^ Using proximal tubule-specific Lrp2-deficient mice, we show that Dapa-mediated preservation of Lrp2 levels, in turn, protects against loss of ApoM, thus attenuating endothelial leak in acute inflammation.

### Study limitations

Our work must be considered in the context of its limitations. First, we utilize an LPS model in both high-fat-fed and chow-fed animals. While the LPS model itself is a generalizable model of cytokine storm, further studies in individual models of infectious vs sterile inflammation will be needed to extend our results to more specific clinical situations. For IVM experiments, we utilized male mice because we examined vascular leak in cremaster venules. Moreover, we performed IVM in lean animals in part because performing these studies in obese animals would add a significant technical challenge and to avoid confounding from genetic interventions on the development of diet-induced obesity. While our work shows conclusively that ApoM is required for the effects of Dapa on vascular leak, there are certainly ApoM-independent effects of Dapa as well.

In addition, our experimental design utilized a pretreatment strategy, which is relevant to patients either already taking an SGLT2i or to those who have recently been started on one. Of note, post hoc analyses of SGLT2i clinical trials have shown lower incidence of infection and sepsis, as well as reductions in mortality due to infections.^6^^,^^62^ However, SGLT2i has not demonstrated survival benefits in clinical trials of either sepsis or COVID-19. This might be explained by the fact that our preclinical results do not support starting an SGLT2i to treat loss of endothelial integrity but instead could apply to a situation where patients potentially at risk could be started on SGLT2i (earlier). Moreover, the results would support a clinically relevant paradigm whereby SGLT2i treatment should not be stopped if the patient experiences an acute inflammatory condition. However, this study does not determine the necessary duration of treatment for SGLT2i to have a protective effect on circulating ApoM levels. Finally, and importantly, our results support a physiological mechanism linking SGLT2i action from the kidney to the vasculature, although we did not identify the precise molecular mechanism by which SGLT2i increases Lrp2. It is formally possible that the effects observed are independent of SGLT2 inhibition and could even be a specific effect of Dapa itself, although there is no clinical evidence to support this concept.

## Conclusions

In summary, our studies in Dapa-treated mice given LPS show that Dapa: 1) preserves levels of Lrp2 in the proximal tubules to therefore prevent loss of ApoM; and 2 preserves endothelial barrier integrity via ApoM/S1P. These observations are translationally relevant and may stimulate future clinical studies of SGLT2i across a variety of disease states. Further translational and clinical studies will be required to determine whether Dapa or other SGLT2i can preserve ApoM levels in disease conditions of significant renal injury.

Perspectives **COMPETENCY IN MEDICAL KNOWLEDGE:** SGLT2is improve cardiorenal outcomes in patients with HF and chronic kidney disease. These drugs are associated with favorable biomarker responses in patients with acute inflammatory conditions such as COVID-19 and reduce vascular leak in preclinical models. The clinical benefits of SGLT2i may be partially related to their effects on vascular leak.

**TRANSLATIONAL OUTLOOK:** In this study, Dapa was associated with increased circulating apolipoprotein M, which was due to an effect on proximal tubular reabsorption through Lrp2, resulting in reduced vascular leak. Future studies should test whether Dapa attenuates acute inflammation and cardiorenal injury in inflammatory conditions such as sepsis.

## Funding support and author disclosures

Dr Javaheri was supported by 10.13039/100000050NHLBI (K08HL138262 and 1R01HL155344), the Children's Discovery Institute of Washington University and 10.13039/100018235St. Louis Children's Hospital (MC-FR-2020-919), the Diabetes Research Center at Washington University in St. Louis of the National Institutes of Health (P30DK020579), the 10.13039/100017553Nutrition Obesity Research Center of the 10.13039/100000002National Institutes of Health (P30DK056341), and the 10.13039/100003085Longer Life Foundation. Dr Guo was supported by the 10.13039/100000968American Heart Association Second Century Early Faculty Independence Award (24SCEFIA125647), 10.13039/100000968American Heart Association Postdoctoral Fellowship Award (898679), and Division of Cardiology at Washington University (GF0012995), as well as the Diabetes Research Center at Washington University in St. Louis of the 10.13039/100000002National Institutes of Health (P30DK020579). Dr Lotfinaghsh was supported by the 10.13039/100000002NIH T32 trainee program (HL007081). The research was in part supported by a research grant from 10.13039/100004325AstraZeneca. The research was, in part, funded by the 10.13039/100000002National Institutes of Health (NIH) Agreement 1OT2HL156812 through the 10.13039/100000050National Heart, Lung, and Blood Institute (NHLBI) CONNECTS program. The views and conclusions contained in this document are those of the authors and should not be interpreted as representing the official policies, either expressed or implied, of the NIH. Dr Javaheri has a pending patent for fusion protein nanodiscs for the treatment of heart failure and eye disease, is a member of the scientific advisory board of Mobius Scientific, and receives research funding from Bitterroot Bio, unrelated to the studies in this manuscript. The present study was in part supported by a research grant from 10.13039/100004325AstraZeneca. Dr Kosiborod receives consulting fees/honoraria from Alnylam, Amgen, Applied Therapeutics, AstraZeneca, Bayer, Boehringer Ingelheim, Cytokinetics, Eli Lilly, Esperion Therapeutics, Janssen, Lexicon, Merck, Novo Nordisk, Pharmacosmos, Sanofi-Aventis, and Vifor Pharma and research grants or contracts from AstraZeneca and Boehringer Ingelheim. Drs Esterline and Oscarsson are employees and stockholders of AstraZeneca. All other authors have reported that they have no relationships relevant to the contents of this paper to disclose.
