# Supplementary material for: Sodium-Glucose Cotransporter Inhibition Preserves Apolipoprotein M During Acute Inflammation in Mice and Humans
Source: JACC Adv. 2025 Jun 25;4(6):101839. doi: 10.1016/j.jacadv.2025.101839 (PMC12277633; doi:10.1016/j.jacadv.2025.101839)
Supplement: Supplemental Material [file mmc1.docx]

**Supplemental Table 1. The sequence of primers used for qPCR.**

| **Species** | **Gene** | **Primer sequence** |
| --- | --- | --- |
| *Mus musculus* | *Apom* | FP: 5'-AACAGACCTGTTCTCCAGCTCG-3'  RP: 5'-GTCCAAGCAAGAGGTCAGAGAC-3' |
|  | *S1pr1* | FP: 5'-ACTACACAACGGGAGCAACAG-3'  RP: 5'-GATGGAAAGCAGGAGCAGAG-3' |
|  | *S1pr2* | FP: 5'-CTCACTGCTCAATCCTGTCATC-3'  RP: 5'-TTCACATTTTCCCTTCAGACC-3' |
|  | *S1pr3* | FP: 5'-TTCCCGACTGCTCTACCATC-3'  RP:5'-CCAACAGGCAATGAACACAC-3' |
|  | *Actb* | FP: 5'-CAGAAGGAGATCACTGCCCT-3'  RP: 5'-AGTACTTGCGCTCAGGAGGA-3' |
|  | *36b4* | FP: 5'-GCTTCGTGTTCACCAAGGAGGA-3'  RP: 5'-GTCCTAGACCAGTGTTCTGAGC-3' |
|  | *Rpl32* | FP: 5'-CCTCTGGTGAAGCCCAAGATC-3'  RP: 5'-TCTGGGTTTCCGCCAGTTT-3' |
|  | *Gapdh* | FP: 5'-ACTCCCACTCTTCCACCTTC-3'  RP: 5'-TCTTGCTCAGTGTCCTTGC-3' |

FP: forward primer; RP: reverse primer

**Supplemental Figures**


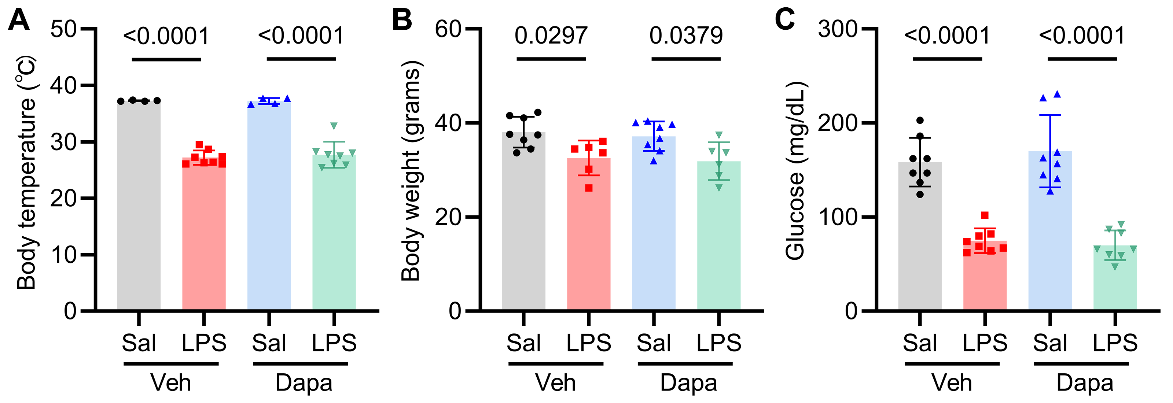


**Supplemental Figure 1. Effects of dapagliflozin on body temperature, body weight, and glucose.** Diet-induced obesity mice were gavaged with vehicle or Dapa (1.25 mg/kg daily for 4 days) before saline or LPS (10 mg/kg IP). **A)** Body temperature; **B)** body weight, and **C)** blood glucose levels were assessed the following morning (n = 4-8/group). All data are presented as mean ± SD. Each dot represents one mouse. Two-way ANOVA with Sidak's correction for multiple comparisons was utilized in panels (A-C).


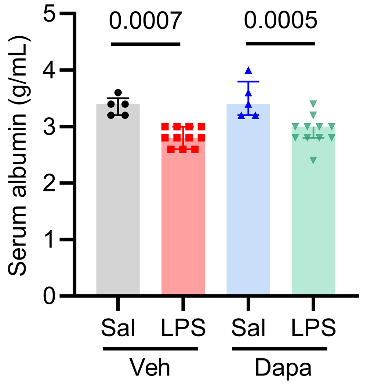


**Supplemental Figure 2. Dapa does not affect the LPS-induced decreases in serum albumin.** Serum albumin levels are shown in diet-induced obesity mice randomized to vehicle or Dapa (1.25 mg/kg gavage daily for 4 days) before saline or LPS (10 mg/kg IP) and euthanized the next day (n = 5-11/group). All data are presented as median ± IQR. Each dot represents one mouse. Two-way ANOVA with Sidak's correction for multiple comparisons was utilized.


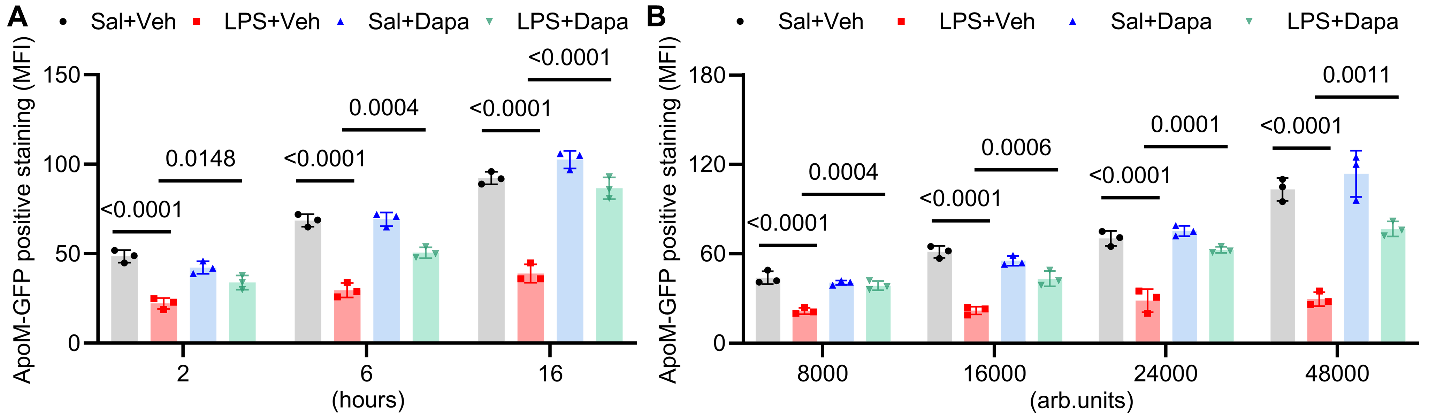


**Supplemental Figure 3.** **Titration on incubation time and amount of ApoM-GFP in HK2 cell experiments.** HK2 cells were grown on 8-chamber slides for 24 hours. The cells were thereafter exposed to Dapagliflozin (6 nM) or vehicle for 16 hours, followed by addition of LPS (0.1 µg/ml) or saline as well as ApoM-GFP. **A)** Quantification of mean fluorescence intensity (MFI) with 24000 arb units of ApoM-GFP over 2, 6, or 16 hours. **B)** Quantification of MFI for further 6 hours with varying concentrations of ApoM-GFP (8000-16000-24000-48000 arb units). n = 3 independent experiments. All data are presented as mean ± SD. Two-way ANOVA with Sidak’s correction for multiple comparison was utilized for each timepoint in (A) and each concentration in (B).


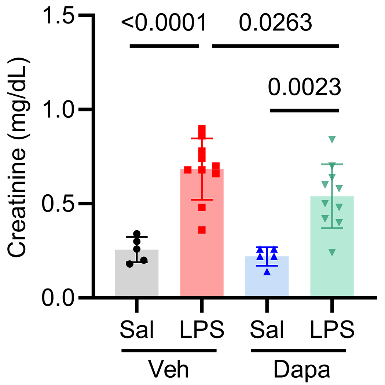


**Supplemental Figure 4.** **Dapagliflozin reduces creatinine in LPS-treated mice.** Diet-induced obesity mice were gavaged with vehicle or Dapa (1.25 mg/kg daily for 4 days) prior to saline or LPS (10 mg/kg IP), measured the day after LPS injection (n = 5-10/group). All data are presented as mean ± SD. Each dot represents one mouse. Two-way ANOVA with Sidak's correction for multiple comparisons was utilized.


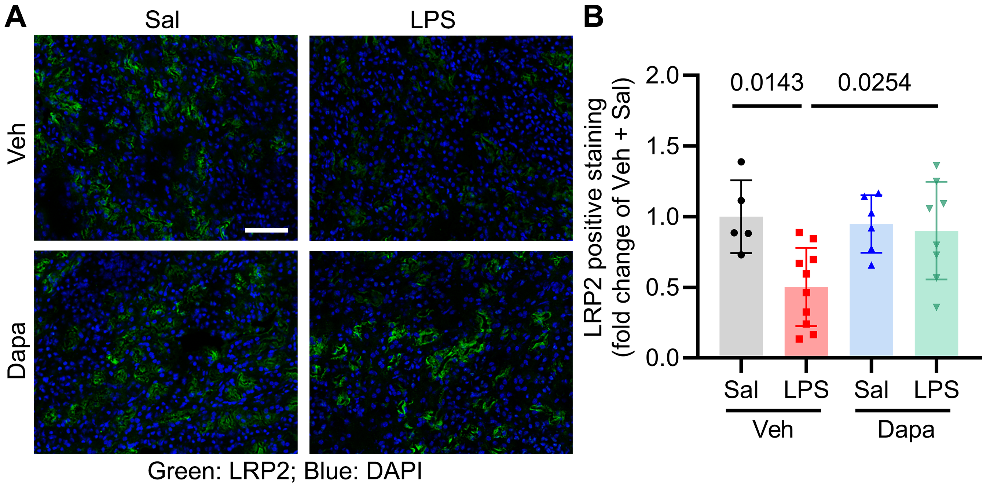


**Supplemental Figure 5. Dapagliflozin preserves circulating ApoM in LPS-treated mice by increasing proximal tubular Lrp2**. Diet-induced obesity mice were randomized to vehicle or Dapa (1.25 mg/kg gavaged daily for 4 days) before saline or LPS (10 mg/kg IP) and euthanized the next day)**. A)** Immunofluorescence staining for Lrp2 (green) and DAPI in kidney frozen sections with quantification of mean fluorescence intensity in **(B**) (n = 5-10/group; scale bar = 50 μm). All data are presented as mean ± SD. Each dot represents one mouse. Two-way ANOVA with Sidak's correction for multiple comparisons was utilized in panel (B).


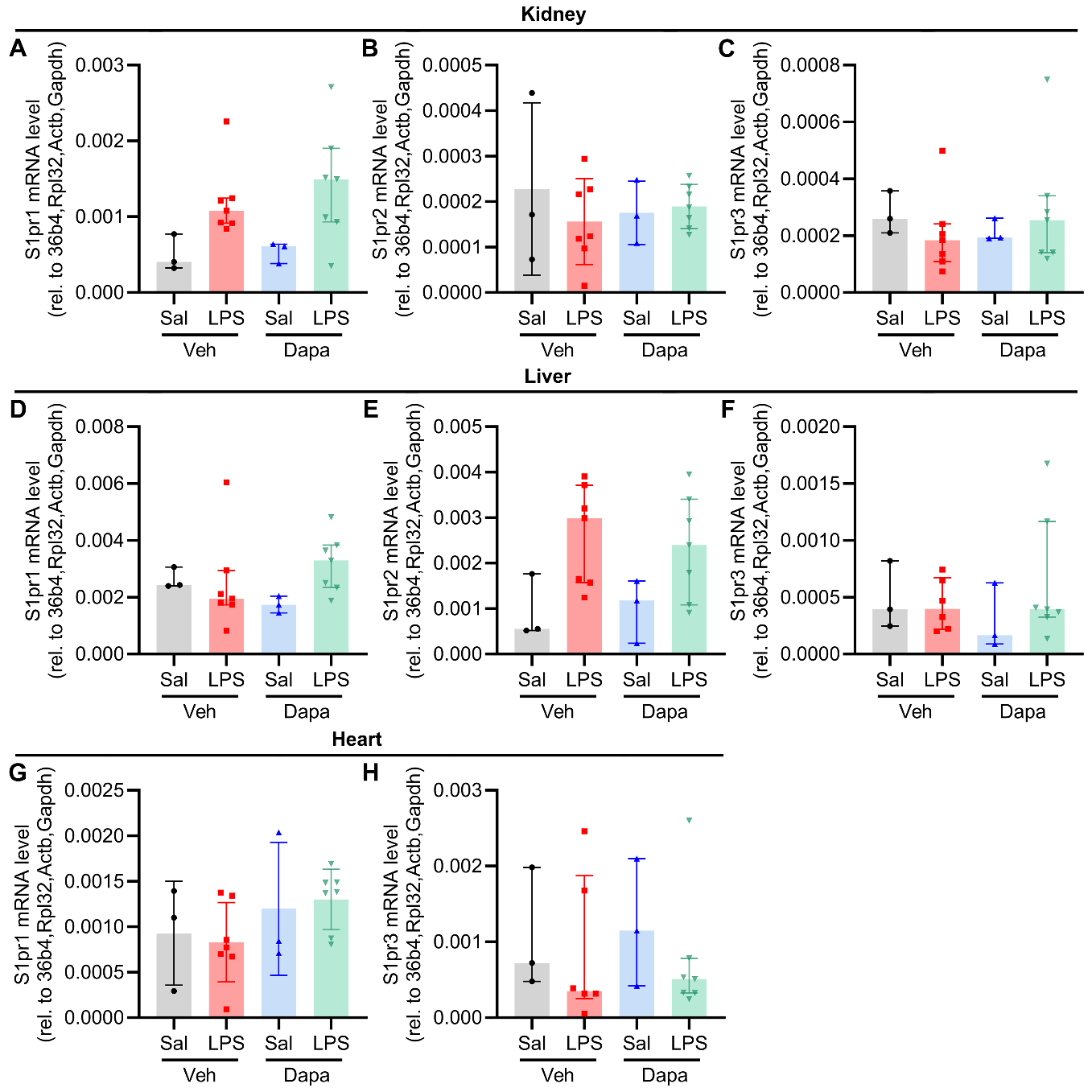


**Supplemental Figure 6. Effects of dapagliflozin on S1P receptor 1, 2, and 3 mRNA abundance.** Diet-induced obesity mice were gavaged with vehicle or Dapa (1.25 mg/kg daily for 4 days) prior to LPS (10 mg/kg IP). Quantification of mRNA abundance in kidney **(A-C)**, liver **(D-F)**, and heart **(G, H)**. n = 3-7/group. All data are presented as mean ± SD or median ± IQR. Each dot represents one mouse. Two-way ANOVA with Sidak's correction for multiple comparisons was utilized.


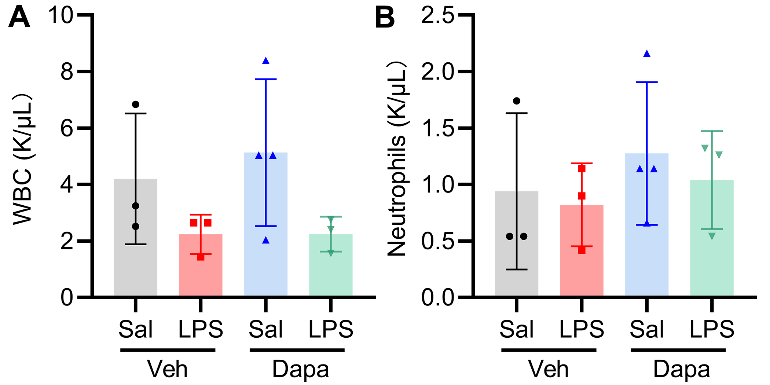


**Supplemental Figure 7. Effects of dapagliflozin on white blood cells.** Diet-induced obesity mice were gavaged with vehicle or Dapa (1.25 mg/kg daily for 4 days) prior to LPS (10 mg/kg IP). Quantification of **A)** total white blood cell count and **B)** neutrophil count. n = 3-4/group. All data are presented as mean ± SD. Each dot represents one mouse. Two-way ANOVA with Sidak's correction for multiple comparisons was utilized in panels (A) and (B).
